# Supplementary material for: The Effect of Subclinical Ketosis on the Peripheral Blood Mononuclear Cell Inflammatory Response and Its Crosstalk with Depot-Specific Preadipocyte Function in Dairy Cows
Source: Animals (Basel). 2024 Jul 6;14(13):1995. doi: 10.3390/ani14131995 (PMC11240650; doi:10.3390/ani14131995)
Supplement: Supplementary file 1 [file animals-14-01995-s001.zip › Supplementary Table S1.pdf]

Supplementary Table S1. Serum metabolic profile of Control and SCK cows

	Control	SCK	SEM	p-value
**BHB (mM)**[1]	0.62	1.30	0.10	<0.01
**NEFA (mM)**[2]	0.46	0.82	0.10	0.02
**Glucose (mM)**	2.72	2.92	0.23	0.53
**Insulin (U/mL)**	7.36	5.10	1.05	0.14
**Cholesterol (mM)**	2.14	1.99	0.27	0.69
**BUN (mM)**[3]	3.96	4.22	0.43	0.67
**Albumin (g/L)**	29.30	31.18	2.45	0.59
**Ca (mM)**	2.05	2.04	0.17	0.97
**P (mM)**	1.68	1.76	0.16	0.74
**Mg (mM)**	0.74	0.77	0.07	0.76
**Na (mM)**	124.40	137.82	9.55	0.32
**K (mM)**	4.09	4.46	0.33	0.42
**Cl (mM)**	87.20	97.36	6.72	0.29

[1]β-hydroxybutyrate; [2]Non-esterified fatty acids; [3]Blood urea nitrogen
