# Supplementary material for: The Effect of Subclinical Ketosis on the Peripheral Blood Mononuclear Cell Inflammatory Response and Its Crosstalk with Depot-Specific Preadipocyte Function in Dairy Cows
Source: Animals (Basel). 2024 Jul 6;14(13):1995. doi: 10.3390/ani14131995 (PMC11240650; doi:10.3390/ani14131995)
Supplement: Supplementary file 1 [file animals-14-01995-s001.zip › Supplementary Table S2.pdf]

Supplementary Table S2. Comparison of Control cows utilized for PBMC isolation and non-ketotic cows used as the preadipocyte source

	Control cows (PBMC source)	Non-ketotic cows (preadipocyte source)	SEM	p-value
**DIM[1]**	8.1	7.0	1.42	0.53
**Lactations**	2.9	3.0	0.77	0.92
**BCS[2]**	3.8	3.7	0.17	0.78
**BHB (mM)[3]**	0.69	0.68	0.06	0.83
**NEFA (mM)[4]**	0.51	0.78	0.13	0.13

[1]Days in milk; [2]Body condition score; [3]β-hydroxybutyrate; [4]Non-esterified fatty acids
